# Catheter Embolization of an Orbital Arteriovenous Fistula in a Patient With Wyburn-Mason Syndrome

**DOI:** 10.7759/cureus.36949

**Published:** 2023-03-31

**Authors:** Balaji Vaithialingam, Swaroop Gopal, Mohammad Sohrab

**Affiliations:** 1 Department of Anaesthesiology, Sakra World Hospital, Bengaluru, IND; 2 Department of Neurosurgery, Sakra World Hospital, Bengaluru, IND

**Keywords:** glue embolization, arteriovenous malformation, arteriovenous fistula, proptosis, wyburn-mason syndrome

## Abstract

A 24-year-old female presented to the emergency department with swelling of the forehead and oculus sinister. A soft, compressible glabellar swelling with proptosis of the oculus sinister was noted on clinical examination. Cerebral angiography revealed a left medial orbital wall arteriovenous fistula with feeders from the left internal maxillary artery, left superficial temporal artery, and left ophthalmic artery. During the cerebral angiography, a diffuse intracranial venous anomaly and left basal ganglia arteriovenous malformations were also noted. A diagnosis of Wyburn-Mason syndrome was made, and the patient underwent catheter embolization of the orbital arteriovenous fistula. After glue embolization of the left external carotid artery feeders, the patient experienced a 50% reduction in glabellar swelling in the immediate postoperative period. Glue embolization of the left ophthalmic artery feeder was planned after six months during the follow-up period.

## Introduction

Wyburn-Mason syndrome (WMS) is a congenital, non-heritable condition characterized by widespread vascular anomalies such as arteriovenous malformations (AVM), arteriovenous fistulas (AVF), and angiomas. The phakomatoses in WMS primarily affect the face, eyes, retina, and brain, with unilateral presentation being more common. WMS is caused by a primitive vascular mesoderm disorder that affects the eyes and mesencephalon. A developmental defect before the seventh week of gestation causes the primitive undifferentiated vascular tissue to persist, resulting in widespread vascular anomalies [1]. WMS has no gender or race preference and commonly manifests in the younger population before the third decade of life. Approximately 105 cases have previously been reported, with the majority of cases being handled conservatively due to high morbidity and mortality. We describe the successful catheter embolization of orbital AVF feeders in a patient with WMS, as well as the technical challenges encountered during the procedure. The patient provided written and informed consent for the publication of this case report.

## Case presentation

**Figure 1:**
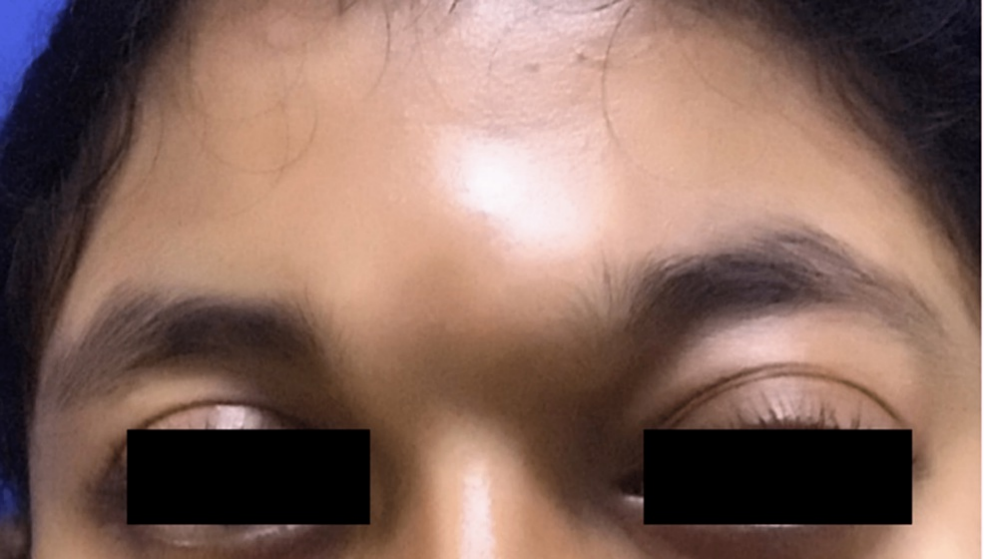
Patient with glabellar swelling and oculus sinister proptosis due to orbital arteriovenous fistula

A 24-year-old female presented with complaints of forehead swelling since birth and gradually progressive oculus sinister (OS) swelling for the previous six months. The patient also had one episode of a generalized tonic-clonic seizure one month back. The clinical examination revealed a 3 x 3 cm soft, non-tender, compressive swelling over the glabella with proptosis of the OS (Figure 1).

**Figure 2:**
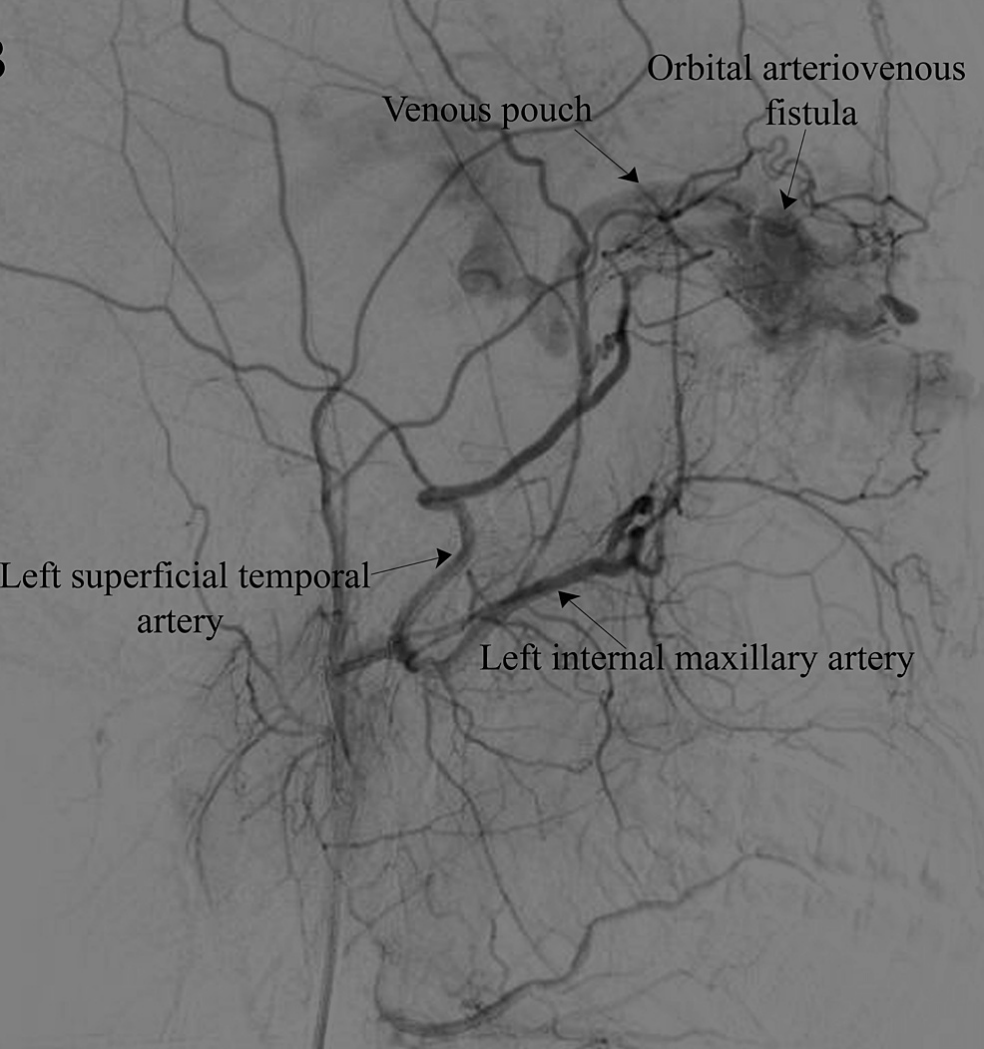
Selective left external carotid angiography (lateral view) showing left maxillary and left superficial temporal artery feeders supplying the left orbital arteriovenous fistula

Auscultation revealed a bruit over the glabellar swelling, and a fundoscopic examination of the OS revealed dilated and tortuous vessels with the normal disc. The patient had a visual acuity of 6/6 and no sensory-motor deficits. In the digital subtraction angiographic suite, a diagnostic cerebral angiography was performed via the femoral route, suspecting a vascular anomaly. Selective angiography of the left external carotid artery (ECA) revealed an AVF in the left medial orbital wall with feeders from the left internal maxillary artery (IMA), left superficial temporal artery (STA), and draining into an anomalous dilated deep vein (Figure 2).

**Figure 3:**
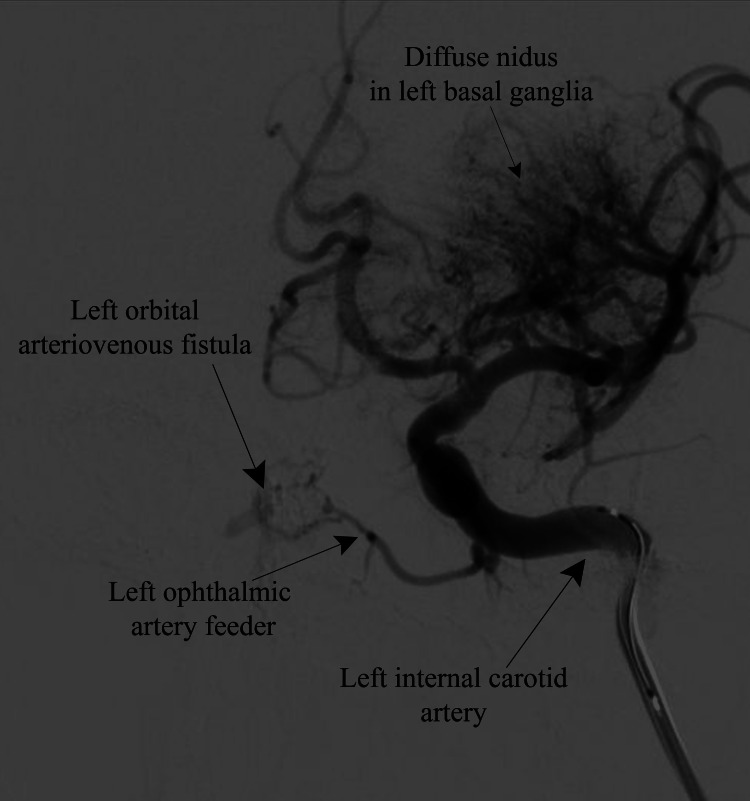
Selective left internal carotid angiography (lateral view) showing the left ophthalmic artery feeder supplying the left orbital arteriovenous fistula and diffuse nidus in the left basal ganglia

Selective angiography of the left internal carotid artery (ICA) revealed an ophthalmic artery feeder supplying the left orbital AVF, as well as diffuse nidus in the left basal ganglia suggestive of AVM (Figure 3).

**Figure 4:**
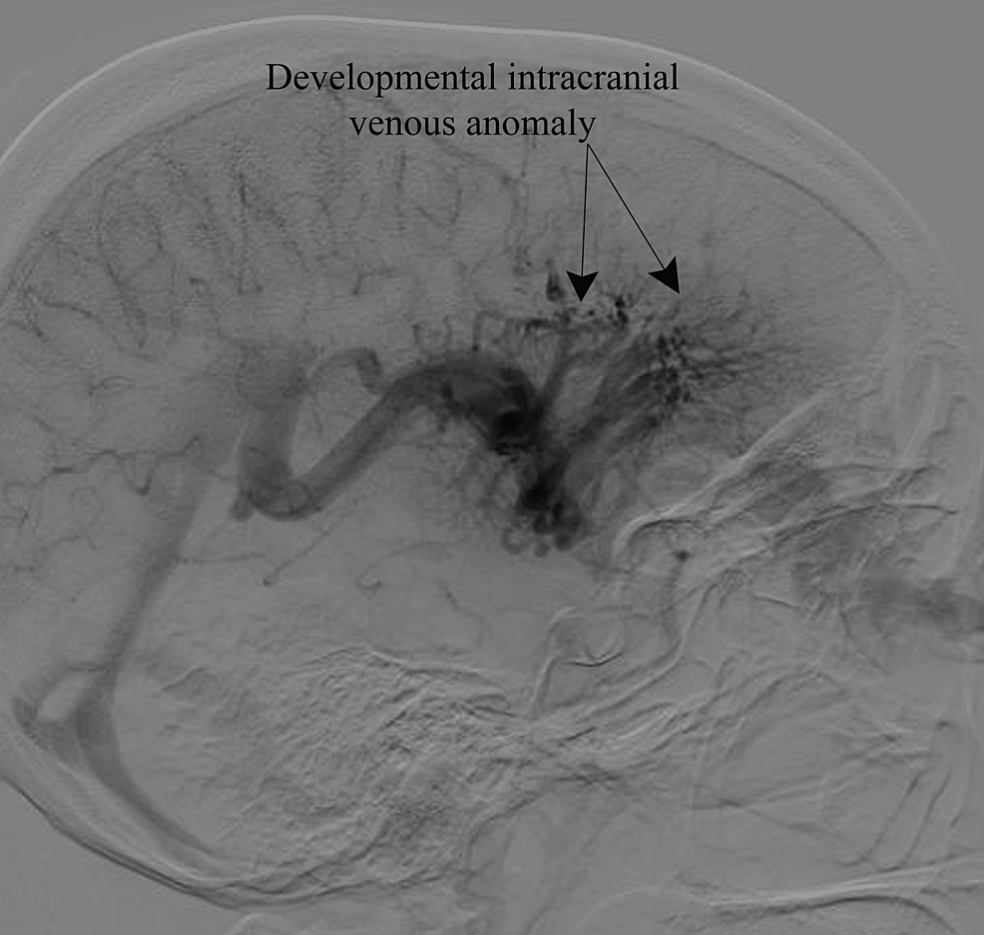
Venous phase of the cerebral angiography (lateral view) showing a diffuse developmental venous anomaly

The left basal ganglia AVM had multiple feeders from the M2 and M3 segments of the middle cerebral artery, with venous drainage into the straight sinus via an anomalous deep vein. During the venous phase of cerebral angiography, diffuse developmental venous anomalies were also observed (Figure 4).

The patient was diagnosed as a case of WMS and selective embolization of the orbital AVF's arterial feeders was planned under general anesthesia, as absolute patient immobility was needed during the procedure. The general anesthesia was induced with 100 mg of intravenous propofol followed by paralysis with 25 mg atracurium. Intravenous fentanyl (100 mcg) was administered to suppress the exaggerated hemodynamic response before tracheal intubation. The 7F right femoral introducer sheath was placed using the Seldinger technique following general endotracheal anesthesia. Intravenous unfractionated heparin (80U/kg) was given to achieve an activated clotting time (ACT) of 250-300. A 5F guiding catheter was introduced coaxially through the femoral introducer sheath and was placed in the left distal ECA. A 1.9F microcatheter with a microwire was inserted into the left IMA, and the feeders were embolized with 1.5 ml of the liquid embolic agent (Squid® 12, Balt Extrusion, Montmorency, France) dissolved in 1.5 ml of dimethyl sulfoxide (DMSO). Following the embolization of the left IMA feeders, the microcatheter was navigated into the left STA, and the feeders were embolized with the liquid embolic agent. A check angiography revealed complete occlusion of the left IMA and STA feeders. Because of technical difficulties in the cannulation of the left ophthalmic artery, the corresponding feeders were not embolized. After ensuring a normal ACT value, the microcatheter with microwire, guiding catheter, and femoral introducer sheath were removed. A compressive bandage was applied to the femoral puncture site after adequate manual compression. After ensuring adequate hemostasis of the femoral puncture site, the general anesthesia was reversed, and the trachea was extubated. The patient was neurologically intact and had a partial reduction in glabellar swelling in the immediate postoperative period. The patient was counseled for glue embolization of the left ophthalmic artery feeder during the second sitting of the follow-up period and was discharged from the hospital.

## Discussion

Wyburn Mason syndrome, also known as cervicofacial arteriovenous metameric syndrome type-2, is a developmental disorder characterized by multiple arteriovenous anomalies that primarily affect the face and brain. Cerebrofacial arteriovenous metameric syndrome (CAMS) is an arteriovenous malformation phenotypic classification system. The hypothalamus, olfactory tract, and corpus callosum are the primary targets of CAMS-1. The maxilla, cheek, cortex, diencephalon, optic nerve, and retina are involved in CAMS-2 (WMS), whereas the mandible, temporal bone, and cerebellum are involved in CAMS-3 [2,3]. Depending on the anatomical location of the vascular lesion, the patient may exhibit a variety of neurological symptoms, including seizure, decreased vision, and hemiparesis. WMS has an uncertain prognosis, and treatment options include conservative management, endovascular glue embolization, surgery, and radiotherapy. Radiotherapy has a high risk of causing endocrine dysfunction, which may be undesirable in young patients. Treatment of vascular anomalies in WMS can result in significant morbidity and mortality due to bleeding, hence patients are often managed conservatively with frequent follow-ups. The management is determined by the anatomical location of the vascular anomaly and the associated symptom. Magnetic resonance imaging can reveal the intracranial localization, extent, and involvement of the eloquent brain area. Digital subtraction angiography can also provide the hemodynamic profile of the vascular malformations and identify the feeder vessels. Only symptomatic patients should be treated, with catheter embolization serving as the primary treatment. Orbital AVF manifests as forehead swelling and proptosis, and treatment is needed in view of cosmesis in young patients. An intracranial AVM is typically treated conservatively, with frequent patient check-ups [4]. An asymptomatic intracranial AVM should be monitored, and intervention may be necessary if the rupture rate exceeds 2.2% per year [5]. Sudden rupture of intracranial arteriovenous malformations can be fatal and necessitates an emergency craniotomy. Catheter embolization necessitates complete immobility, so general anesthesia is required. Squid® liquid embolic agent (Balt Extrusion, Montmorency, France) is an ethylene vinyl alcohol copolymer that is dissolved in DMSO with suspended micronized Tantalum powder and comes in two formulations (Squid® 12 and Squid® 18). Squid® 12 has a homogeneous percolation into the vasculature due to its lower viscosity and is commonly preferred. Squid® 12, on the other hand, can block the vasa nervorum due to its excellent diffusion properties, resulting in cranial nerve palsies [6]. During the procedure, the patient should be adequately heparinized, and transient systemic hypotension can prevent distal glue migration during the glue deposition process. Distal glue migration, arterial dissection, intracranial bleeding, and arterial thrombosis are all possible complications during catheter embolization.

## Conclusions

WMS is a rare congenital disorder that can cause life-threatening bleeding due to widespread vascular malformations and necessitates a multidisciplinary approach. Asymptomatic vascular malformations should be monitored, and only symptomatic patients should be treated. Endovascular catheter embolization is less invasive and has fewer complications than other treatment options.
